# Altered Nuclear Functions in Progeroid Syndromes: a Paradigm for Aging Research

**DOI:** 10.1100/tsw.2009.159

**Published:** 2009-12-16

**Authors:** Baomin Li, Sonali Jog, Jose Candelario, Sita Reddy, Lucio Comai

**Affiliations:** ^1^ Department of Molecular Microbiology and Immunology, Keck School of Medicine, University of Southern California, Los Angeles, USA; ^2^ Department of Biochemistry and Molecular Biology, Institute for Genetic Medicine, Keck School of Medicine, University of Southern California, Los Angeles, USA

**Keywords:** aging, Werner syndrome, Hutchinson-Gilford progeria syndrome, lamin, telomere

## Abstract

Syndromes of accelerated aging could provide an entry point for identifying and dissecting the cellular pathways that are involved in the development of age-related pathologies in the general population. However, their usefulness for aging research has been controversial, as it has been argued that these diseases do not faithfully reflect the process of natural aging. Here we review recent findings on the molecular basis of two progeroid diseases, Werner syndrome (WS) and Hutchinson-Gilford progeria syndrome (HGPS), and highlight functional connections to cellular processes that may contribute to normal aging.

## INTRODUCTION

As the average life span of the human population is steadily rising, at least in the western world, the increased incidence of a wide range of diseases with age has become a critical human health issue and an important topic in biomedical research. The identification and biochemical characterization of the molecular alterations that occur during the aging process can provide critical insights into the cellular events that predispose us to the development of age-related diseases. Progeroid syndromes, which are rare disorders that shorten life expectancy, represent a valuable model for studying the aging process, as patients with these diseases display early onset of many, although not necessarily all, features of normal aging. The identification, in the last decade, of the genetic mutations responsible for two progeroid diseases, Werner syndrome (WS) and Hutchinson-Gilford progeria syndrome (HGPS), has permitted molecular studies aimed at dissecting the underlying causes of these diseases and their possible relationship with the process of natural aging. These studies, which have provided a number of critical information at the molecular and cellular levels on specific nuclear functions that, when altered, cause WS and HGPS, could perhaps provide valuable information for understanding the molecular basis of age-associated pathologies in the general population.

## THE MOLECULAR BASIS OF WERNER SYNDROME

### Werner Syndrome Protein is a RecQ Helicase with a Unique Exonuclease Activity

WS is an autosomal-recessive, premature aging disorder[1,2] that is typically diagnosed in individuals 20–30 years of age. WS patients display a striking predisposition to an early onset of several diseases that are generally observed during normal aging, such as cataracts, arteriosclerosis, osteoporosis, type II diabetes mellitus, and a variety of tumors, primarily of mesenchymal origin. Myocardial infarction and cancer are the most common causes of death among WS patients, with a median age of death of approximately 47 years[1,3]. WS is caused by mutations in a single gene located on chromosome 8[4]. The gene encodes a protein, termed Werner syndrome protein (WRN), which belongs to a class of enzymes known as RecQ helicases[4,5].

WRN helicases can unwind a number of different DNA structures, from linear duplex DNA to a 5' single-strand region of flap DNA substrates, synthetic replication forks, and Holliday junctions[6,7]. In addition, WRN as well as Bloom syndrome protein (BLM), a related human RecQ helicase, can efficiently unwind G-rich DNA substrates that have the tendency to form structures known as G quadruplexes, which consist of four arrangements of guanine stabilized by Hoogsteen hydrogen bonding and monovalent cations[6]. G-rich sequences are widely distributed within the human genome and are found at the promoter region of several proto-oncogenes, at immunoglobulin switch regions, ribosomal DNA gene locus, and telomeric repeat regions[8]. WRN is unique among the members of the RecQ family of helicases in that it possesses a 3' to 5'exonuclease activity that is highly homologous to the nuclease domain of *E. coli* DNA polymerase I and ribonuclease D (RNase D)[9]. Since the helicase properties of WRN are similar to those of BLM, it is likely that the characterization of the exonuclease activity and identification of its *bona fide* substrates will provide mechanistic insights on the specific cellular function of the WRN protein.

All the WRN mutations that have been identified to date in WS patients are nonsense or frameshift mutations leading to the synthesis of a truncated protein lacking the nuclear localization signal, which is located at the carboxyl-terminal end of WRN. As most cell lines from WS patients show no detectable WRN protein[10], the prevailing hypothesis is that the truncated protein fails to enter the nucleus and is rapidly degraded, thus resulting in complete loss of WRN function. At the cellular level, cultured primary fibroblasts from WS patients have a decreased replicative life span compared to fibroblasts from healthy individuals, and display chromosomal translocation and deletions[11,12,13], suggesting that lack of WS function causes genome instability. In an effort to create animal models of the disease, several groups have generated mice with WRN mutations. Mice bearing a deletion of the helicase domain or a point mutation that eliminates WRN expression do not show signs of premature aging, genomic instability, or increased sensitivity to genotoxins[14,15]. In contrast, several of the phenotypes observed in WS patients are recapitulated in late-generation mice lacking both WRN and telomerase activities[16,17], suggesting that defects in telomere length homeostasis may contribute to the pathology of the disease, at least in mice.

### WRN in the Metabolism of DNA Ends

To gain insights into the physiological function of WRN, our lab and others have pursued studies aimed at identifying molecular partners of WRN and characterizing their functional relationship. These studies have revealed binary interactions between WRN and several nuclear proteins, including Ku70/80, BLM, FEN1, TRF2, p53, pol$\beta$, pol$\delta$, RAD51, RAD52, RAD54B, RPA, POT1, PCNA, PARP1, MRE11, and Cdc5L[18,19,20,21,22,23,24,25,26,27,28,29,30,31,32]. As these proteins are involved in a variety of nuclear processes, including DNA replication, repair, recombination, and telomere metabolism, these findings suggest that WRN might function in any of these DNA transactions. Among this plethora of interacting proteins, the physical and functional interaction between WRN and the Ku heterodimer has been the focus of several investigative studies since it is likely to play a key role in processes required for maintaining genome stability[18,19,33,34,35,36,37]. Indeed, Ku70/80 is a heterodimer (Ku) that binds DNA ends and functions in the repair of DNA double-strand breaks (DSBs) by nonhomologous DNA end joining (NHEJ)[38]. In addition, Ku has recently been implicated in the maintenance of telomeres through as-yet-incompletely-defined mechanisms[39]. Hence, it is likely that the interaction between WRN and Ku influences processes occurring at both pathological (DNA breaks) and natural (telomeres) DNA ends (Fig. 1). A subpopulation of Ku forms a stable functional complex with WRN in human cells and is responsible for the recruitment of WRN to DNA ends and for altering the properties of WRN exonuclease activity on a variety of DNA substrates *in vitro*[18,28,35,36,37]. In support of the concept that the interaction between WRN and Ku plays an important role in cell function, genetic studies demonstrated that cells from Ku80-null mice display similarities to human WS fibroblasts, including a high degree of genomic instability characterized by chromosome translocations and rearrangements[40,41,42]. Significantly, both Ku70 and Ku80 knockout mice display characteristics of premature aging[41,43]. Thus, biochemical and genetic evidence strongly suggest that Ku and WRN participate in a common, but yet to be defined, cellular pathway, possibly linked to the metabolism of DNA ends, which regulates cellular and organismal aging.

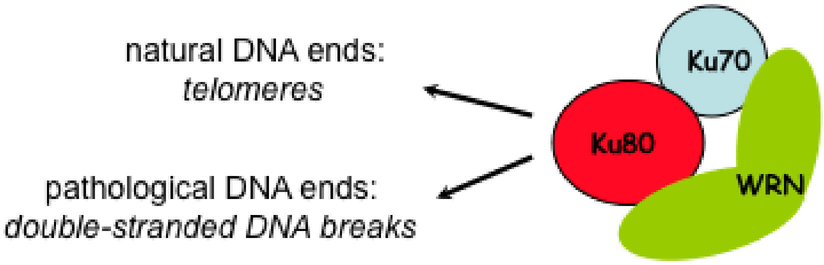

**FIGURE 1.** The identification of a strong interaction between WRN and the Ku70/80 heterodimer suggests a role for WRN in the metabolism of DNA ends.

## Abnormal Telomere Homeostasis is an Important Molecular Event in WS Pathology

Recent studies have demonstrated that a subpopulation of WRN localizes at telomeres (Fig. 2; [44,45]) and interacts with at least two telomere-associated proteins, TRF2 and POT1[26,27,46]. *In vitro* studies have further indicated that POT1 stimulates WRN helicase[27] and limits WRN exonuclease processivity on telomeric substrates with 3' overhangs[47]. In addition, the telomere repeat binding factor TRF2 has been shown to regulate both WRN enzymatic activities, although the specific effect that TRF2 exerts on WRN exonuclease has not been conclusively established[45,46]. Critical evidence supporting a functional role for WRN at telomere comes from cell-based studies, which demonstrated that lack of WRN leads to sporadic loss of telomeric DNA generated by lagging DNA synthesis (sister telomere loss or STL), a process that can be prevented by expression of either a fully functional or exonuclease-deficient WRN, but not by a WRN mutant lacking helicase activity[44]. This finding suggests that helicase, but not exonuclease, activity is important for preventing STL. Other studies have further indicated that WRN may play a role in the preservation of telomeres in cells that maintain telomere length by a telomerase-independent, recombination-based mechanism termed "Alternative Lengthening of Telomere" (ALT)[45,48]. Collectively, these studies point to telomeres as at least one important *bona fide* substrate of WRN and suggest that telomere dysfunction contributes to the pathology of WS.

Human telomeres are composed of several kilobases of the repetitive hexamer TTAGGG and contain a 3' single-stranded DNA extension that is thought to invade the complementary strand and establish the protective terminal structure termed telomeric-loop (T-loop) (Fig. 3)[49,50,51]. Disruption of the T-loop activates a DNA damage-response pathway and leads to cell cycle arrest, cell senescence, or apoptosis[52,53,54,55].

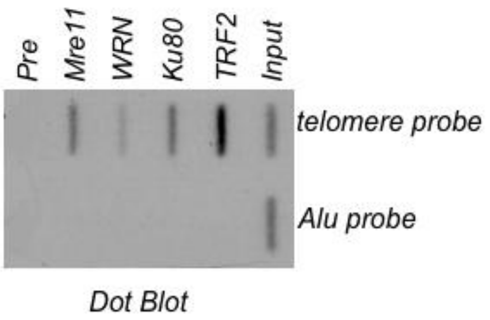

**FIGURE 2.** Chromatin immunoprecipitation assays demonstrate the association of WRN with telomeres. Human fibroblasts were fixed with formaldehyde to stabilize protein-DNA complexes. The chromatin was then mechanically sheared to generate small DNA fragments. These fragments were immunoprecipitated with antibodies specific to the indicated proteins (pre = preimmune serum). Following immunoprecipitation, the cross-link was reversed, DNA was isolated and analyzed by slot blot hybridization using either telomeric or Alu repeats probes.

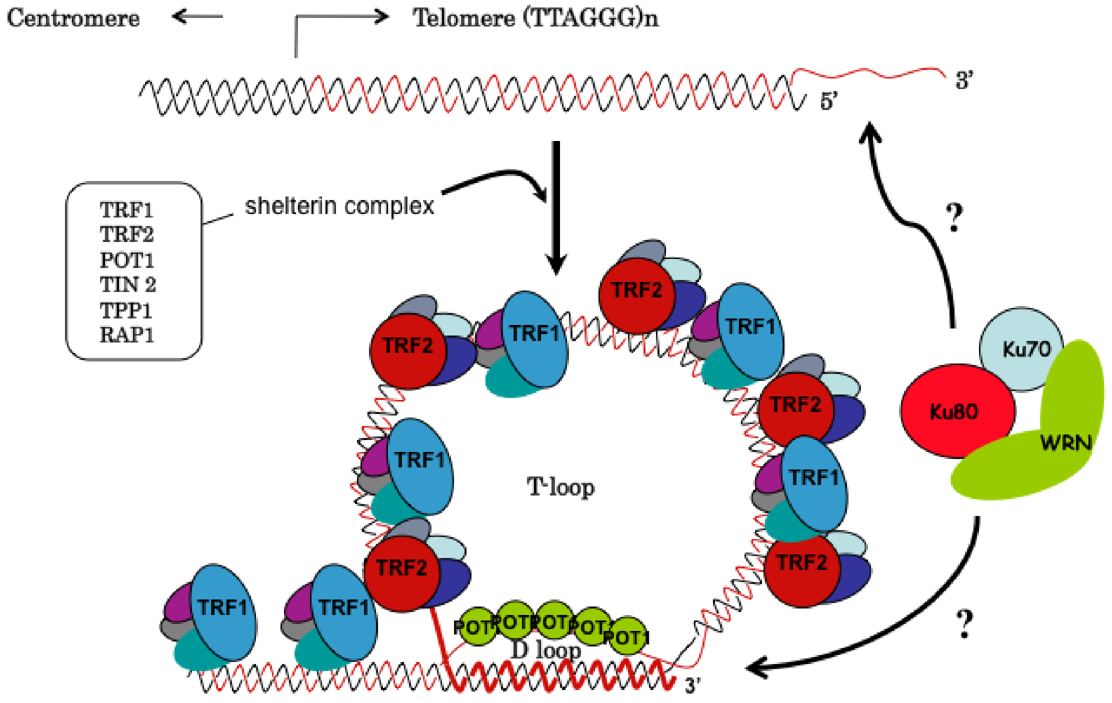

**FIGURE 3**. Hypothetical model of telomere end protection. The shelterin complex is required for the formation of the protective T-loop and plays a critical role in preventing telomere dysfunction leading to apoptosis or senescence. The mechanism by which the WRN complex influences the metabolism of chromosome ends is poorly understood.

The formation and maintenance of the T-loop is mediated by a multiprotein complex termed shelterin, which includes telomeric DNA-binding proteins, such as telomere repeat factor 1 and 2 (TRF1 and TRF2), the single-stranded telomeric DNA-binding protein POT1, and TIN2, TPP1, and RAP1, three factors that do not bind to telomeric DNA directly (Fig. 3)[56]. The shelterin complex is implicated in the control of telomere length and protects telomere termini from end-to-end fusion and recombination[56]. Alterations that disturb the stoichiometry or composition of this complex can lead to dysfunctional telomere, and profoundly affect cell function and survival. Indeed, overexpression of a mutant TRF2 lacking the amino terminal basic domain ($TRF2^{\Delta B}$) causes abrupt telomere deletions, formation of extrachromosomal telomere circles, and cellular senescence[57]. Significantly, WRN is required for $TRF2^{\Delta B}$-mediated telomere shortening and cell senescence[58], suggesting a functional interplay between WRN and shelterin in the maintenance of the proper telomeric structure. Unexpectedly, elevated levels of t-circles are also observed in telomerase-positive WS cells and their level is not enhanced by expression of $TRF2^{\Delta B}$, suggesting that loss of WRN function *per se* can induce the production of these aberrant telomere structures[58]. Helicase and exonuclease activities are required to suppress the formation of t-circles in cells expressing telomerase, suggesting that both activities play an important role in regulating telomere topology.

Extrachromosomal DNA circles composed of telomeric or nontelomeric sequences have been detected in cancer cells and during aging of mammalian cells[59,60,61]. Moreover, an increase in extrachromosomal ribosomal DNA (rDNA) circles has been reported as a hallmark of replicative aging in yeast[62]. Although the contribution of telomeric DNA circles to the development of cancer or to the aging process in mammals is unknown, these extrachromosomal DNA structures can potentially be a source of genome instability. For example, t-circles could integrate into intrachromosomal loci, thus generating internal telomeric repeats that are genetically unstable. The presence of extrachromosomal telomeric repeats could also have an indirect effect on telomere function by sequestering telomere-binding proteins away from their chromosomal binding sites, thus leaving telomeres in danger of nucleolytic attack. It is also possible that t-circles could be utilized as templates for telomerase-independent telomere elongation, thus contributing to telomere length maintenance. Indeed, t-circles have been detected in human cancer cells that maintain telomere length through the telomerase-independent process ALT, and the formation of t-circles in ALT cells, as wells as cells expressing $TRF2^{\Delta B}$, requires the presence of the recombination factors XRCC3 and NBS1[57,63]. In contrast to ALT cells, down-regulation of XRCC3 does not reduce t-circle formation in WS cells, suggesting that alternative mechanisms of t-circle formation operate in these cells[58]. Notably, t-circles have also been observed in cells from Pot1 knockout mice, supporting the concept that alterations in telomere termini protection can lead to the formation of these extrachromosomal DNA structures[64]. Whether t-circles are simply by-products of aberrant telomere metabolism or directly contribute to the maintenance of telomere length in any of these settings is currently unclear.

Remarkably, the WRN-interacting partner Ku70/80 has also been detected at telomeres and implicated in the regulation of telomere maintenance in a range of organisms including yeast, plants, mice, and humans[39]. Ku has been proposed to function both in the protection of telomere ends from degradation and in the regulation of telomere length, possibly through a functional interaction with the telomerase holoenzyme[65,66,67]. Yet, how Ku operates at telomeres seems to differ among species as, for example, cells from Ku knockout plants show extreme telomere elongation, while yeast lacking Ku display loss of telomere repeats[68,69]. While the basis for this divergence in function is unknown, it is likely to reflect natural species-specific variations in telomere biology. Interestingly, a recent study has demonstrated that inactivation of Ku, which functionally interacts with WRN-like activities both in human and plants[18,70], induces the formation of t-circles in *Arabidopsis*[71]. Importantly, t-circle formation in Ku-deficient plants is not suppressed by inactivation of several genes involved in the homologous recombination pathway, including XRCC3[71]. Although this phenotype is reminiscent of that observed in WS fibroblasts, it remains to be determined whether Ku plays any role in the repression of t-circles in human cells (see note below)[*].

[*] A recent study has shown that Ku is required to repress the formation of t-circles in telomerase-positive human cells[123].

## THE MOLECULAR BASIS OF HUTCHINSON GILFORD PROGERIA SYNDROME

### Alterations in Prelamin A Processing Result in a Cellular Progeroid Phenotype

HGPS is a rare, but devastating, autosomal-dominant genetic disorder observed in about 1 in 4 million children worldwide, which is associated with an early onset of several pathological features typical of premature aging[72,73]. The disease is characterized by severe growth retardation, micrognathia, abnormal dentition, osteoporosis and bone hypoplasia, gonadal defects, lipodystrophy, atherosclerosis, type 2 diabetes, and skeletal muscle atrophy[74]. Children with HGPS typically die due to cardiac failure between the ages of 13 and 16 years. It was not until 2003 that the long-sought genetic mutation responsible for the devastating disease HGPS was found to be located within the gene coding for lamin A[75,76]. Lamin A is a nuclear protein and a member of the family of intermediate filaments. It is synthesized as a precursor molecule (prelamin A), which is then sequentially processed into the mature lamin A protein (Fig. 4). Processing involves a transient farnesylation and carboxymethylation of the carboxyl-terminal tail, which is ultimately cleaved off by the zinc metalloprotease ZMPSTE24. The processing time in normal human cells has been determined to take approximately 3 h[77,78].

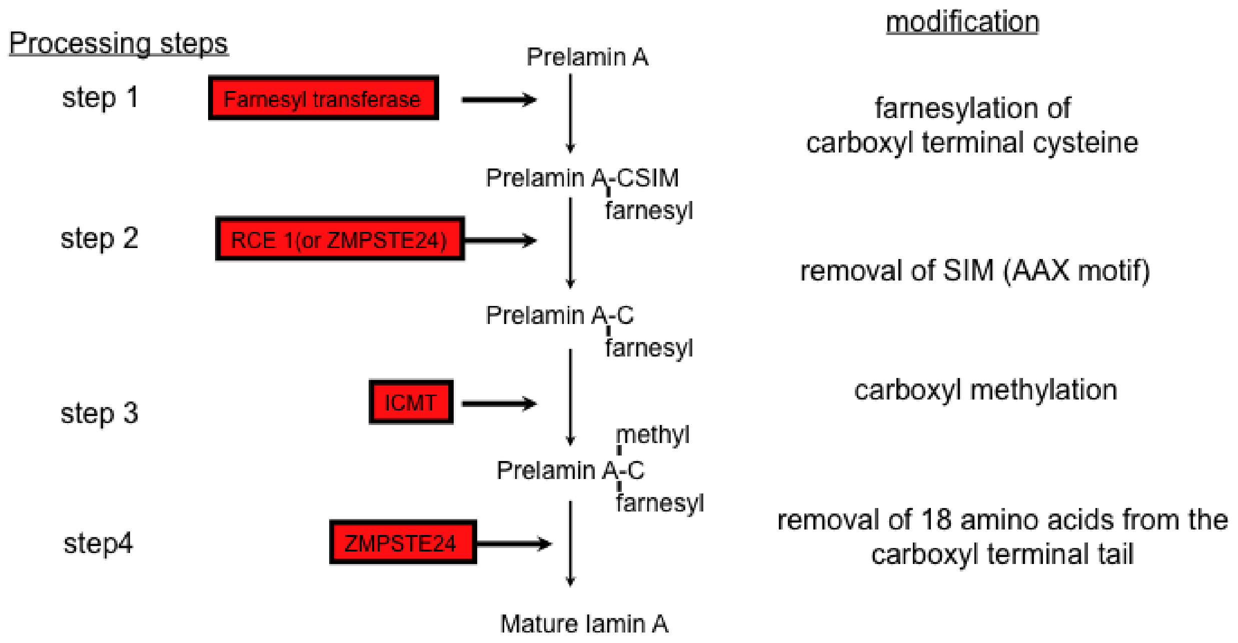

**FIGURE 4.** Schematic representation of the prelamin A processing pathway. Newly synthesized prelamin A is modified by a farnesyltransferase that adds a 15-carbon farnesyl moiety to the carboxyl terminal cysteine (CSIM; step 1). Farnesylated prelamin A is then modified by a prenyl-CAAX-specific endoprotease (AAX = aliphatic-aliphatic-any amino acid) termed RAS converting enzyme 1 (RCE1; step 2), which removes the last three amino acids (SIM) at the carboxyl terminus of the protein. The metalloprotease ZMPSTE24 may also be involved in the removal of these three amino acids. This prelamin A intermediate is then the substrate for isoprenylcysteine carboxyl methyltransferase (ICMT; step3), which methylesterifies the carboxyl terminal farnesylated cysteine. Lastly, farnesylated lamin A is processed into mature, unfarnesylated lamin A by ZMPSTE24, which cleaves the carboxyl terminal 15 amino acids of the protein (step 4).

The most common mutation in HGPS patients is a *de novo* heterozygous C to T transition at nucleotide 1824 of the *LMNA* gene, which results in the production of a cryptic splice site within exon 11[75,76]. Translation of the misspliced mRNA produces a mutant lamin A protein with an internal deletion of 50 amino acids, which lacks the internal proteolytic cleavage site necessary to remove the farnesylated carboxyl-terminal tail to generate mature lamin A[75,76]. The genetics of HGPS suggest that the mutant lamin A protein, termed progerin, acts as in a dominant-negative fashion. Progeria cells and normal fibroblasts or Hela cells expressing ectopic progerin display severe growth defects and altered nuclear membrane morphology, and inhibition of prelamin A prenylation by treatment with farnesyltransferase inhibitors (FTIs) reverses, in part, the growth properties and the nuclear morphology defects of these cells[79,80,81,82,83].

Striking evidence on the critical role that lamin A plays, not only in the pathology of progeria, but more generally in human health, comes from a relatively large number of studies that have demonstrated that specific point mutations in the *LMNA* gene cause a range of human disorders that affect distinct tissues or organs and are collectively known as laminopathies[84,85,86]. These include Emery-Dreifuss muscular dystrophy (EDMD2/3), Limb-Girdle muscular dystrophy (LGMD1B), dilated cardiomyopathy (conduction-system disease, CMD1A), familial autosomal-dominant partial lipodystrophy (Dunnigan variety, FPLD), autosomal-recessive Charcot-Marie-Tooth disease (DMT2B1), mandibuloacral dysplasia (MAD), and atypical WS. As the molecular basis of these diseases remains to be defined, it is anticipated that a detailed analysis of the cellular function of lamin A will provide key mechanistic insights on the causes of certain tissue-specific diseases.

## Lamin A is an Organizer of Chromatin Structure and Regulator of Gene Expression and DNA Replication

The mechanisms by which mutations in lamin A alter nuclear function and cause disease are unclear. Lamin A is a component of the nuclear lamina, which is located in the inner side of the nuclear membrane and is believed to provide a mechanical framework for the support of the nuclear envelope[87,88]. In addition to this structural role, lamin A has been proposed to play an important function in the regulation of nuclear processes, since it interacts with chromatin, either directly or through association with other proteins[87,89,90], and binds to at least three transcription factors: the serum responsive element binding protein (SREBP1), the retinoblastoma transcription factor (RB), and activating protein 1 (AP1)[91,92,93]. These findings suggest that lamin A may be involved in the organization of chromatin structure and regulation of gene expression, and one or both of these functions may be affected by the presence of mutant lamin A, including progerin. Indeed, accumulation of progerin causes relocalization or decreased levels of markers of heterochromatin, including heterochromatin protein 1$\alpha$ (HP1$\alpha$), histone H3 trimethylated on lysine 9 (H3K9-3me), and histone H3 trimethylated on lysine 27 (H3K27-3me), loss of heterochromatin associated with the inactive X chromosome, and activation of genes located in the normally silenced pericentric regions of the chromosomes[94,95,96,97,98]. Significantly, treatment with farnesyltransferase inhibitors, a condition that ameliorates the cellular phenotype, increases the pattern of histone H3K9-3me in HGPS cells[97]. Evidence for a direct involvement of lamin A in regulation of gene expression comes from studies showing that expression of a dominant-negative lamin A mutant lacking the NH2-terminal domain inhibits RNA polymerase II activity in both mammalian cells and isolated nuclei from *Xenopus laevis*[99]. Significantly, the gene expression profile of progeria cells closely resembles that of fibroblasts from old-age individuals[100], and comparison of the transcriptome between HGPS and control fibroblasts led to the identification a large number of genes that are expressed differentially in HGPS cells[101]. Moreover, the analysis of genes whose expression is altered soon after ectopic expression of progerin has indicated that aberrant activation of several genes involved in Notch signaling pathway may be implicated in the pathogenesis of progeria[102].

The interaction between lamin A and chromatin may also play a regulatory role in DNA synthesis. Indeed, lamin A has been shown to localize with sites of DNA replication and directly interacts with proliferating cell nuclear antigen (PCNA)[103,104]. Moreover, studies in cultured cells expressing a mutant form of lamin A lacking the amino terminal domain demonstrated abnormal distribution of two elongation factors, proliferating cell nuclear antigen (PCNA) and replication factor complex (RFC), and altered elongation phase of DNA replication[99,105]. Alterations in DNA replication have not been reported in progeria cells yet. However, it could be surmised that defective DNA replication leading to the collapse of replication forks may be at the basis of the increased levels of damaged DNA observed in progeria cells[106]. Thus it will be important to determine whether a fundamental nuclear process such as DNA replication is adversely affected by expression of progerin.

## The Lamin A Pathway in Cellular and Organismal Aging

Although the relationship between HGPS and natural aging remains unclear, the recent identification of small amounts or progerin produced by sporadic usage of the cryptic slice site in cultured cells and skin biopsies from healthy individuals has raised the prospect that age-dependent accumulation of this mutant lamin A protein may be implicated in cell senescence and general human aging[107,108,109]. Significantly, the treatment of fibroblasts from old individuals with reagents designed to specifically reduce expression of the progerin-coding mRNA results in the reversal of age-associated nuclear defects, suggesting a direct causal relationship between the levels of this lamin A variant and these phenotypes[108]. A potential functional link between the lamin A pathway and cellular aging is further underscored by studies that demonstrated that in the absence of disease-causing mutations, alterations in the normal lamin A metabolism triggered by increased expression of wild-type prelamin A negatively influence cell function[83,110]. Notably, a mild increase in the expression of wild-type prelamin A in normal fibroblasts is sufficient to induce, albeit with slower kinetics, the set of alterations observed in progeria cells, including growth defects, nuclear membrane abnormalities, cell death, and premature senescence[83]. These alterations are reversed by overexpression of ZMPSTE24, indicating that abnormal processing of wild-type prelamin A is responsible for these phenotypic changes. An implication of this finding is that possible age-associated decreases in the activity of ZMPSTE24 could have a major impact on the processing of prelamin A and ultimately accelerate the aging process.

Expression of progerin induces aberrant, nonuniform thickening of the nuclear lamina and a reduced extractability of lamin A from nuclei, indicating a redistribution and aggregation of lamin A from the nucleoplasm to the nuclear membrane[96]. Significantly, the aberrant pattern of lamin A localization seen in progeria cells is also observed in cells expressing elevated levels of wild-type lamin A, in cells from old-age individuals, and in senescent cells, but rarely observed in cells from young individuals[83,111]. Whether these changes in lamin A localization are causally involved in cellular aging remains to be determined.

The role of lamin A in eliciting age-associated pathologies at the organismal level has been investigated by generating transgenic and knockout mouse models. *Lmna* knockout mice are normal at birth, but quickly develop a muscle phenotype that resembles Emery-Dreifuss muscular dystrophy[112]. Mice with a targeted mutation that unexpectedly alters *Lmna* splicing develop progeroid features, including loss of subcutaneous fat, decreased bone density, heart pathology, and muscle atrophy[113]. Two murine models of HGPS have been generated by the use of either a BAC clone containing the human mutation or by replacement of a 3’ region of *Lmna* gene with a single exon that encodes the carboxyl terminus of progerin[114,115]. The BAC transgenic mice show a mild phenotype, which is restricted to the smooth muscle cells of the vascular system, while the second mice model displays several progeria-like phenotypes, including severe growth retardation, abnormal dentition, kyphosis, and fragile bones. However, the second mice model does not demonstrate vascular defects, cardiomyopathy, gonadal defects, muscle atrophy, and weakness, which also characterize progeria patients. Intriguingly, eliminating the synthesis of mature lamin A reduces disease phenotypes in mice carrying an HGPS allele[116], possibly suggesting a detrimental interplay between the normal and the mutant lamin A. It is currently unclear why the entire spectrum of defects observed in HGPS patients is not observed in either mouse model. Significantly, the relevance of the lamin A processing pathway in the development of age-associated pathologies has been underscored by the demonstration that mutations in the *ZMPSTE24* gene, both in humans and mice, lead to progeria-like syndromes characterized by growth retardation, skeletal abnormalities, and cutaneous atrophy[117,118,119].

## CONCLUDING REMARKS

The identification of the mutations responsible for the premature aging diseases WS and HGPS has prompted intense investigations on the molecular basis of these diseases and their potential relationship to normal aging. These studies have shown that both mutations affect the function of proteins that participate in nuclear processes required for maintaining proper cell function. Although at first sight the pathways affected by these progeroid diseases appear unrelated, a potentially interesting link is emerging, as recent studies have revealed that altered telomere homeostasis is observed not only in WS as a pathological phenotype, but also in progeria cells and cells ectopically expressing progerin[110,120]. Interestingly, expression of the catalytic subunit of the reverse transcriptase telomerase (TERT), which rescues the premature senescence of WS cells, reverts the limited growth phenotype of HGPS cells, although it is unclear whether this is mediated by a telomere-lengthening process or involves noncanonical functions of telomerase[121]. The link between telomere metabolism and lamin A is further emphasized by studies showing that lamin A is required for maintaining the proper spatial organization, chromatin structure, and length of telomeres[111,122]. The precise relationship between progeroid syndromes and telomere homeostasis, as well as the impact of altered WRN or lamin A function during normal aging, are works in progress. It is anticipated that these studies will provide mechanistic insights for the development of therapeutic agents for a wide range of age-associated diseases that impact distinct organs and tissues.

## ACKNOWLEDGMENTS

We thank members of the Comai and Reddy labs for valuable discussions. The studies on WS and HGPS in our lab have been supported by the National Institutes of Health (R01AG023873), the Zumberge Innovation Fund, and the Progeria Research Foundation, and were conducted in a facility constructed with support from the Research Facilities Improvement Program Grant Number C06 RR014514-01, C06 RR10600-01, and C06 CA62528 from the National Center for Research Resources, National Institutes of Health. J.C. is supported by an NIH Predoctoral Fellowship (F31 GM076861).
